# Response of reef corals on a fringing reef flat to elevated suspended-sediment concentrations: Molokaʻi, Hawaiʻi

**DOI:** 10.7717/peerj.699

**Published:** 2014-12-18

**Authors:** Paul L. Jokiel, Kuʻulei S. Rodgers, Curt D. Storlazzi, Michael E. Field, Claire V. Lager, Dan Lager

**Affiliations:** 1 University of Hawaiʻi, Hawaiʻi Institute of Marine Biology, Kāneohe, HI, USA; 2 United States Geological Survey, Pacific Coastal and Marine Science Center, Santa Cruz, CA, USA; 3 Hawaiʻi State Division of Aquatic Resources, Honolulu, HI, USA

**Keywords:** Sediment, Coral reefs, Hawaiʻi

## Abstract

A long-term (10 month exposure) experiment on effects of suspended sediment on the mortality, growth, and recruitment of the reef corals *Montipora capitata* and *Porites compressa* was conducted on the shallow reef flat off south Molokaʻi, Hawaiʻi. Corals were grown on wire platforms with attached coral recruitment tiles along a suspended solid concentration (SSC) gradient that ranged from 37 mg l^−1^ (inshore) to 3 mg l^−1^ (offshore). Natural coral reef development on the reef flat is limited to areas with SSCs less than 10 mg l^−1^ as previously suggested in the scientific literature. However, the experimental corals held at much higher levels of turbidity showed surprisingly good survivorship and growth. High SSCs encountered on the reef flat reduced coral recruitment by one to three orders of magnitude compared to other sites throughout Hawaiʻi. There was a significant correlation between the biomass of macroalgae attached to the wire growth platforms at the end of the experiment and percentage of the corals showing mortality. We conclude that lack of suitable hard substrate, macroalgal competition, and blockage of recruitment on available substratum are major factors accounting for the low natural coral coverage in areas of high turbidity. The direct impact of high turbidity on growth and mortality is of lesser importance.

## Introduction

Sedimentation is a major detrimental factor on coral reefs (Johannes, 1975; Cortes & Risk, 1985; Grigg & Birkeland, 1997). Extreme sedimentation can smother and kill corals (Edmondson, 1928), whereas suspended sediment reduces irradiance, restricts photosynthesis, reduces coral growth (Davies, 1991; Anthony & Connolly, 2004), and impedes coral larval survival and development (Gilmour, 1999). Pollutants that accompany land-derived sediment can further affect settlement, recruitment, and survivorship of coral and larvae (Glynn et al., 1986).

The classic work of Rogers (1983) and Rogers (1990) concludes that mean suspended-particulate matter for reefs that are not impacted directly by human activities generally is less than 10 mg l^−1^. Concentrations above this value result in fewer coral species, less live coral, lower coral growth rates, greater abundance of branching forms, reduced coral recruitment, decreased calcification, decreased net productivity of corals, and slower rates of reef accretion. Erftemeijer et al. (2012) found that reported tolerance limits of coral reef systems for chronic suspended sediment concentrations range from <10 mg l^−1^ in pristine offshore reef areas to >100 mg l^−1^ in marginal near shore reefs. Some coral species can tolerate short-term exposure (days) to suspended sediment concentrations as high as 1,000 mg l^−1^ while others show mortality after exposure (weeks) to concentrations as low as 30 mg l^−1^.

Quantifying the mechanisms responsible for coral reef decline in high sediment areas has been more challenging. As pointed out by Fabricius (2005), the mechanisms by which sediment limits coral development are complex and are difficult to isolate and quantify. Erftemeijer et al. (2012) showed a significant relationship of coral sensitivity to turbidity and sedimentation with growth form and note that some of the variation in sensitivities reported in the literature may have been caused by differences in the type and particle size of sediments involved.

Weber, Lott & Fabricius (2006) conducted short term (12–60 h) coral exposures to ten different sediment types at environmentally relevant concentrations (33–160 mg dry weight cm^−2^) in laboratory and field experiments. Changes in the photosynthetic yield of the coral *Montipora peltiformis* was measured by pulse–amplitude modulated chlorophyll fluorometry (PAM) as proxy for photophysiological stress from exposure, and to determine rates of recovery. Different sediments exerted greatly contrasting levels of stress in the corals. Grain size and organic and nutrient-related sediment properties were key factors determining sedimentation stress in corals after short-term exposure. Photophysiological stress was measurable after 36 h of exposure to most of the silt-sized sediments, and coral recovery was incomplete after 48–96 h recovery time. The four sandy sediment types caused no measurable stress at the same concentration for the same exposure time. Stress levels were strongly related to the values of organic and nutrient-related parameters in the sediment, weakly related to the physical parameters, and unrelated to the geochemical parameters measured. *M. peltiformis* removed the sandy grain size classes more easily than the silt, and nutrient-poor sediments were removed more easily than nutrient rich sediments. They found that silt-sized and nutrient-rich sediments can stress corals after short exposure, while sandy sediments or nutrient-poor silts affect corals to a lesser extent.

These results were followed up by Weber et al. (2012) who postulated that the coral death was microbially mediated. Microsensor measurements were conducted in mesocosm experiments and in naturally accumulated sediment on corals. Organic-rich sediments caused tissue degradation within 1 d, whereas organic-poor sediments had no effect after 6 d. In the harmful organic-rich sediment, hydrogen sulfide concentrations were low initially but increased progressively because of the degradation of coral mucus and dead tissue. Dark incubations of corals showed that separate exposures to darkness, anoxia, and low pH did not cause mortality within 4 d. However, the combination of anoxia and low pH led to colony death within 24 h. Their data suggest that sedimentation kills corals through microbial processes triggered by the organic matter in the sediments, and that the organic enrichment of coastal sediments is a key process in the degradation of coral reefs exposed to terrestrial runoff.

**Figure 1:**
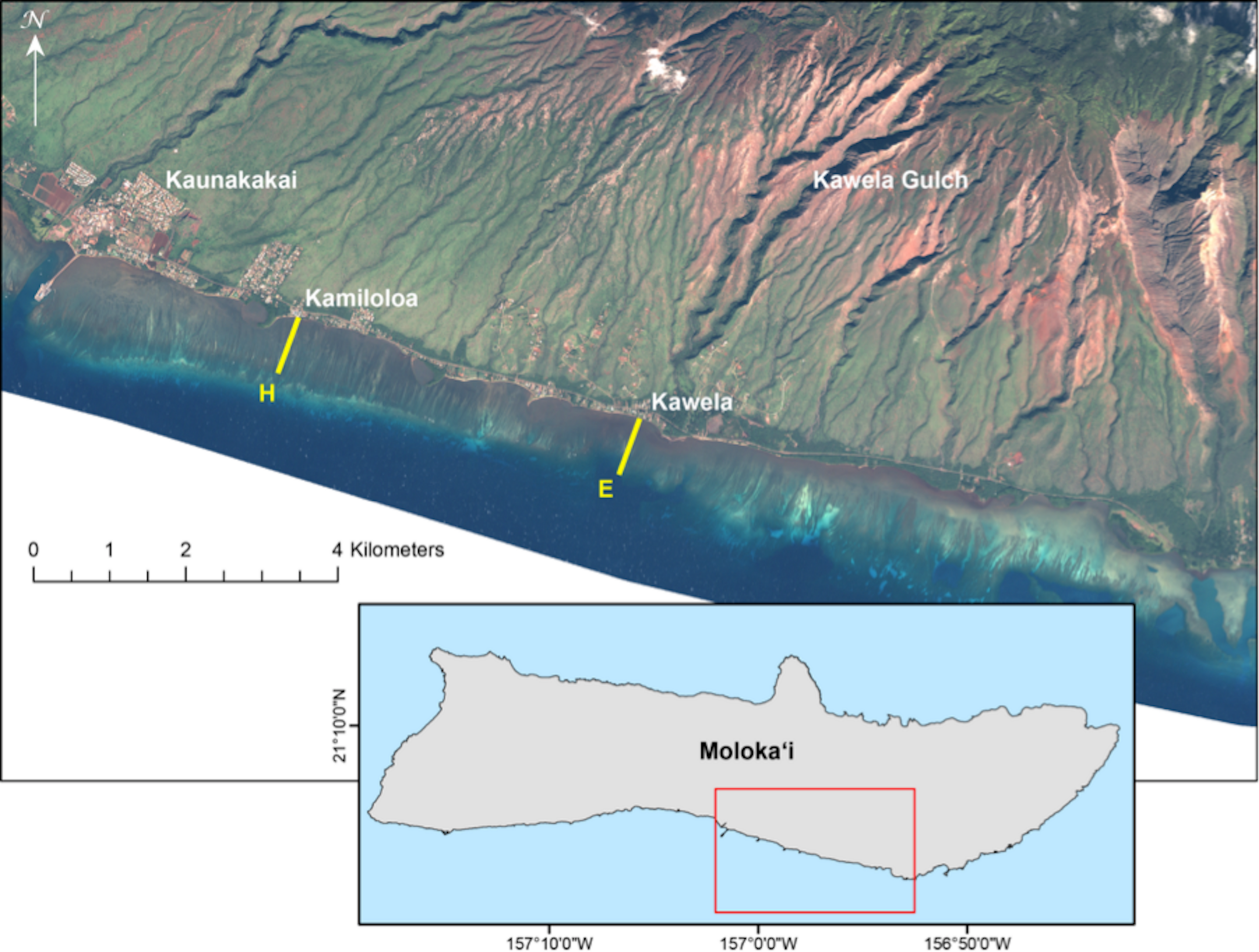
Landsat satellite image of study site on the south shore of Molokaʻi, Hawaiʻi. Landsat satellite image showing locations of U.S. Geological Survey water-quality transects on south Molokaʻi reef flat (transect E at Kawela, and transect H at Kamiloloa) where coral growth platforms were established for this study. Note the strong turbidity gradient along the coast that is clearly visible from the air.

Results of short-term exposure of corals to sedimentation are useful in identifying acute effects. However, from a demographic point of view it is critical that we gain a better understanding of coral population dynamics in relation to impacts from sediment on reef ecosystems. Therefore, we undertook a study to measure the impact of suspended particulate matter on coral recruitment, growth, and mortality for the two locally dominant reef flat coral species along a gradient of terrigenous sediment impact (Fig. 1).

## Materials and Methods

### Site description

An ideal site to study the effects of sediment on corals is located on the south coast of Molokaʻi in the Hawaiian Islands (Fig. 1). This is an extensive shallow reef flat with depths of approximately 0.5 m to 1.0 m from inshore to offshore locations. Extensive geological, oceanographic, sedimentological, and biological data are available from this area (see synthesis by Field et al., 2008a, and references therein), and provide a solid basis for the design of this biological study. A strong inshore to offshore turbidity gradient occurs at all points along this coast due to extensive terrigenous sediment discharge from multiple drainage basins (Field et al., 2008b), of which Kawela is the most significant (Fig. 1). The USGS program established nine transects with six water-sampling stations (50 m, 100 m, 250 m, 400 m, 550 m, 700 m from shore) along each cross-shore transect on the Moloka‘i reef flat in 2004 to describe the physical and sedimentological conditions.

### Methods

At the USGS stations during 7–9 April 2007, water samples were collected daily using a Niskin water sampler just above the seabed along two shore-normal transects (E and H) at a series of six fixed stations along each transect and located 50 m (sites E-50 and H-50), 100 m (sites E-100 and H-100), 250 m (sites E-250 and H-250), 400 m (sites E-400 and H-400), 550 m (sites E-550 and H-550) and 700 m (sites E-700 and H-700) from shore on the reef flat (Fig. 1). These data were used to evaluate the spatial and temporal variability in suspended-sediment concentration (SSC) in mg l^−1^ as defined and measured by Presto et al. (2006). The SSC measurement was used in this study because it is an established USGS protocol for collection of sediment and for analysis of suspended sediment samples (Edwards & Glysson, 1999). The water samples were processed following methodology described in Poppe et al. (2000). This SSC data was used in site selection of two transects that showed a distinct sediment gradient increasing with distance from shore. Transect E, offshore Kawela, is directly impacted from sediment emanating from Kawela Gulch. Transect H, off Kamiloloa, is to the west and downcoast from the large sediment inputs farther east (Fig. 1), but is still subject to elevated SSCs. Biological survey data collected at each site (Rodgers et al., 2005) were used as criteria in site selection to determine abundance and distribution of corals along a sediment gradient. In addition, biological and physical survey data were collected at each site including benthic cover, coral diversity and richness, and sediment composition and grain-size. A total of 40 digital photos were taken within the 7 m radius at each of the 54 stations (Rodgers et al., 2005). Our operating hypothesis was that a gradient in coral mortality, growth, and recruitment would be detected along the turbidity gradient.

**Figure 2:**
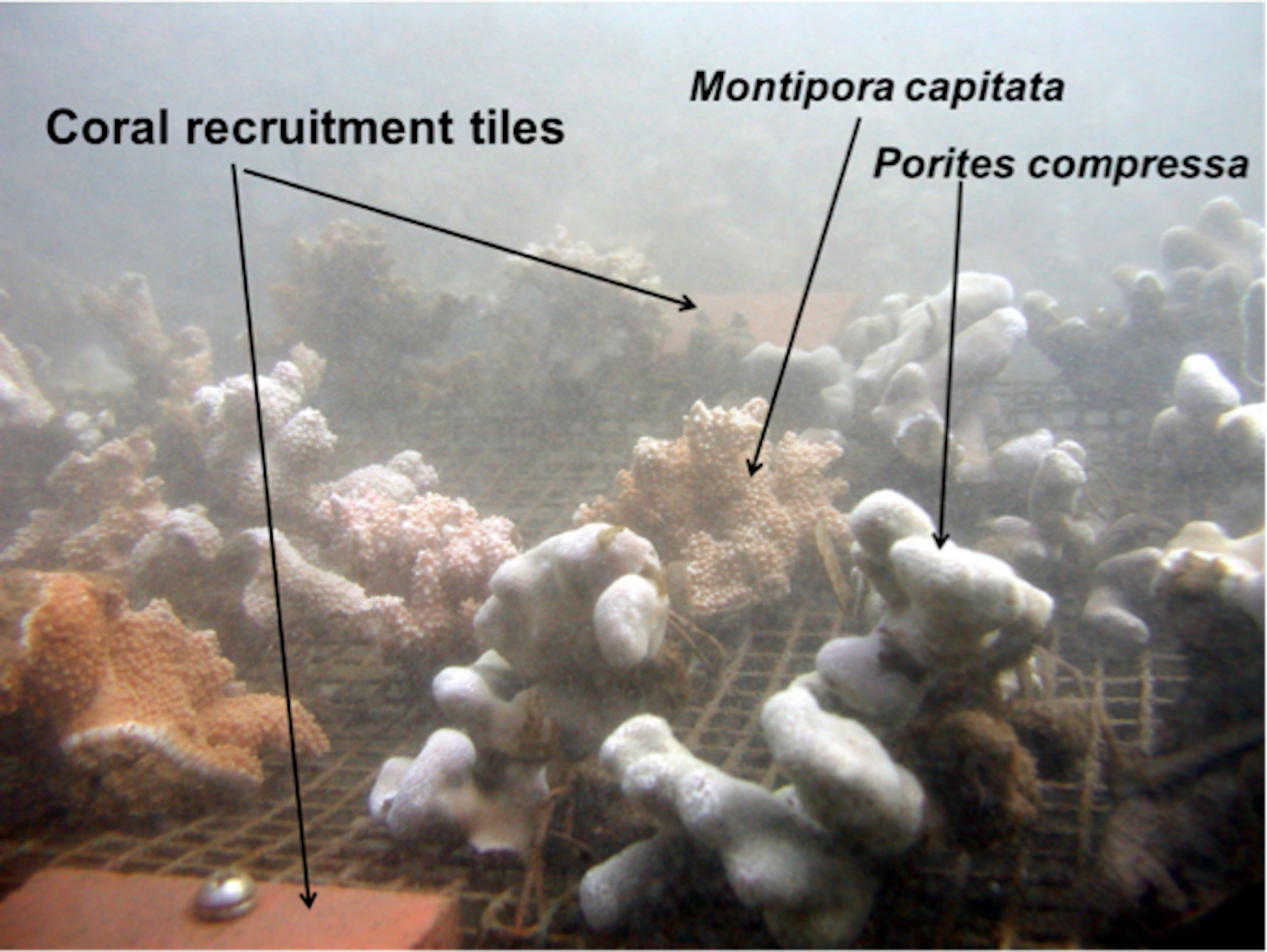
Photograph of coral growth wire platform with corals (*n* = 40) and coral recruitment tiles (*n* = 4).

A coral experimental setup consisting of a coral growth wire platform and two standard coral recruitment arrays (Brown, 2004) were established at 10 of the 12 USGS water sampling stations along Transects E and H (Fig. 2) at five stations: 100 m, 250 m, 400 m, 550 m, and 700 m from shore. The USGS station at 50 m from shore was not selected due to the shallow depth at low tide and lack of coral growth.

Two species of corals (*Montipora capitata* and *Porites compressa*) dominate the south Moloka‘i reef flat and thus were chosen for the study. Small colonies approximately 10 cm in diameter were collected in the area and labeled with embossed plastic tags attached to the coral with vinyl-coated wire. The initial skeletal weight of each colony was measured using the buoyant weighing technique (Jokiel, Maragos & Franzisket, 1978). Changes in coral growth were determined by subtracting the final buoyant weights of each coral from its initial weight in grams. Twenty corals of each species were randomly assigned to the various positions on each platform placed at the ten stations (*n* = 400). Each platform held 20 *P. compressa* and 20 *M. capitata* colonies. The corals were attached to the vinyl-coated wire-mesh coral growth platform with fine vinyl coated wire. Each platform was anchored to the bottom with steel rods to prevent movement and maintain the corals slightly above the reef substratum. In some of the shoreward locations, corals would sink into soft mud if placed directly on the bottom. Each frame held two pairs of standard coral recruitment tiles extensively used as a preferred substrate for scleractinian recruitment for a number of studies around the world: Australia (Harriott & Fisk, 1987; Mundy, 2000), French Polynesia (Adjeroud, Penin & Carroll, 2007), the Caribbean (Kuffner et al., 2006), the Red Sea (Abelson, Olinky & Gaines, 2005), and here in Hawaiʻi (Friedlander & Brown, 2005). A set of two unglazed terracotta tiles (10 cm × 10 cm surface area of 0.023 m) was assembled with a plexiglass spacer to create a gap between the two tiles. This gap was created to inhibit grazing by invertebrates and fishes on the underside of tiles where most coral recruitment occurs (Birkeland, Rowley & Randall, 1981; Adjeroud, Penin & Carroll, 2007; Cox & Ward, 2003; Arnold & Steneck, 2011). One set was placed at either end of the growth platform (Fig. 2). Retrieved tiles were properly labeled with site, location, plate number, and orientation (top or bottom). Tiles were then rinsed and dried. Recruits were counted using a Wild Heerbrugg M5 zoom stereo dissecting microscope. All surfaces of each tile were scanned (top, bottom, and sides). Coral recruits were circled with a fine-tipped permanent marker and initial taxonomic assignment made and recorded in a spreadsheet. Coral recruits were identified using descriptions and photographs provided by Dr. Eric Brown of the National Park Service. Since juvenile corals have few useful taxonomic characters (Babcock et al., 2003), coral recruits were identified to genus according to differences in morphology. The experiment was set out on 2 February 2007 and on 24 October 2007 the corals were brought back into the laboratory. Mortality, partial mortality, and changes in skeletal weight were determined for each coral and the number of coral recruits on the coral recruitment tiles was recorded. Mortality and partial mortality were recorded in three categories: live (complete living tissue), partial (fractional living tissue), and mortality (no living tissue). All macroalgae was removed from each platform, rinsed, dried and an ash-free dry weight was determined.

General linear models (GLM) were selected through backward elimination using *α* = 0.05 in R (R Development Core Team, 2008). GLMs were conducted using growth and mortality/partial mortality as dependent variables regressed against the predictors: species, turbidity, distance from shore, and macroalgae to explore the variation in coral growth and mortality.

## Results and Discussion

The full GLM models included growth and mortality as the response variables, and the main effects of coral species, site, distance from shore, turbidity, and benthic cover of macroalgae. The best model in explaining coral growth included species, distance from shore, and benthic cover of macroalgae (*R*^2^ = 66.91%, *p*-value = 0.0001). The model that best explains mortality/partial mortality includes species, distance, macroalgae, turbidity, and the interaction between species and distance and the interaction between species and turbidity (*R*^2^ = 60.39%, *p*-value = 0.0038).

### Suspended-sediment concentration (SSC) gradients

USGS data (Field et al., 2008b) showed that the suspended sediment off south-central Moloka‘i along the transects E (Kawela) and H (Kamiloloa) ranged from coarse sand to coarse silt that was moderately to very poorly sorted. The sediment varied in composition from approximately 25–90% carbonate, and thus 10–75% terrigenous volcanics, by mass, with the fine-grain silts and clays being predominantly terrigenous in origin. The percentage of terrigenous sediment decreased with distance from shore while the percentage carbonate material increased with distance from shore. The percentage organic carbon in the samples averaged 8.1 ± 2.6% by mass, suggesting that the SSC values presented here may be on the order of 5–12% less than a comparative measure of TSS presented in mass per volume. Turbidity data (*n* = 6, 657) decreased by 76% between the landward E-100 and seaward E-400 sites, showing a similar pattern to the decrease in SSCs (81%) between the same sites. Marquis (2005) points out that turbidity and suspended sediment are not the same. Suspended sediment refers to particulate matter moved by water and is typically measured in milligrams of particulate matter to liters of water. Particles greater than 50 µm (i.e., sand) will fall out of the water column in seconds once the water is calmed. Silt-sized particles (50–2 µm) can remain in suspension for minutes in still water, while clay-sized particles (<2 µm) can remain in suspension indefinitely. Turbidity is a measure of the cloudiness of water and is usually quantified in nephelometric turbidity units (NTUs). Either organic matter, such as algae, or inorganic particles, like silt, can cause turbidity. Turbidity measurements in the field can be taken rapidly. At a given site there is a strong relationship between turbidity and suspended sediment (Marquis, 2005). Therefore, turbidity measurements along the south Moloka‘i coast show the same patterns as the SSC data and reinforce our observations of a strong sediment gradient. Suspended sediment concentrations at the experimental sites are presented in Table 1.

**Table 1:** Suspended sediment concentration (SSC) values taken at the 12 experimental sites.

Transect	Distance mark (m)	Mean mg l^−1^	SE
E	50	37.2	8.6
E	100	36.8	6.0
E	250	23.1	4.7
E	400	7.9	2.1
E	550	3.2	0.7
E	700	4.5	1.8
H	50	31.1	7.2
H	100	24.1	9.8
H	250	10.2	2.8
H	400	6.0	1.4
H	550	6.1	1.5
H	700	3.1	0.4

**Figure 3:**
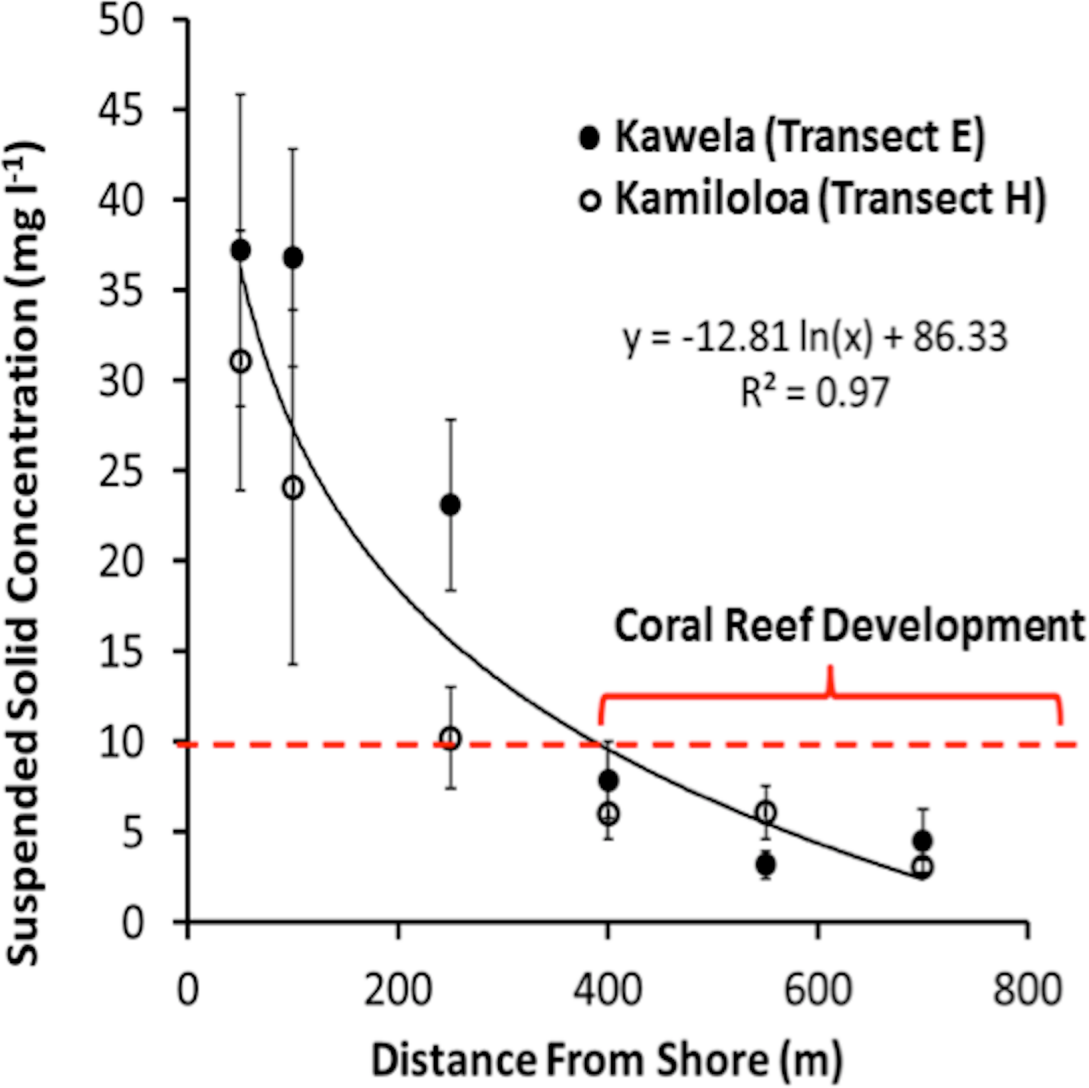
Suspended-sediment concentration (SSC) ±SE along Kawela (E) and Kamiloloa (H) transects.

**Figure 4:**
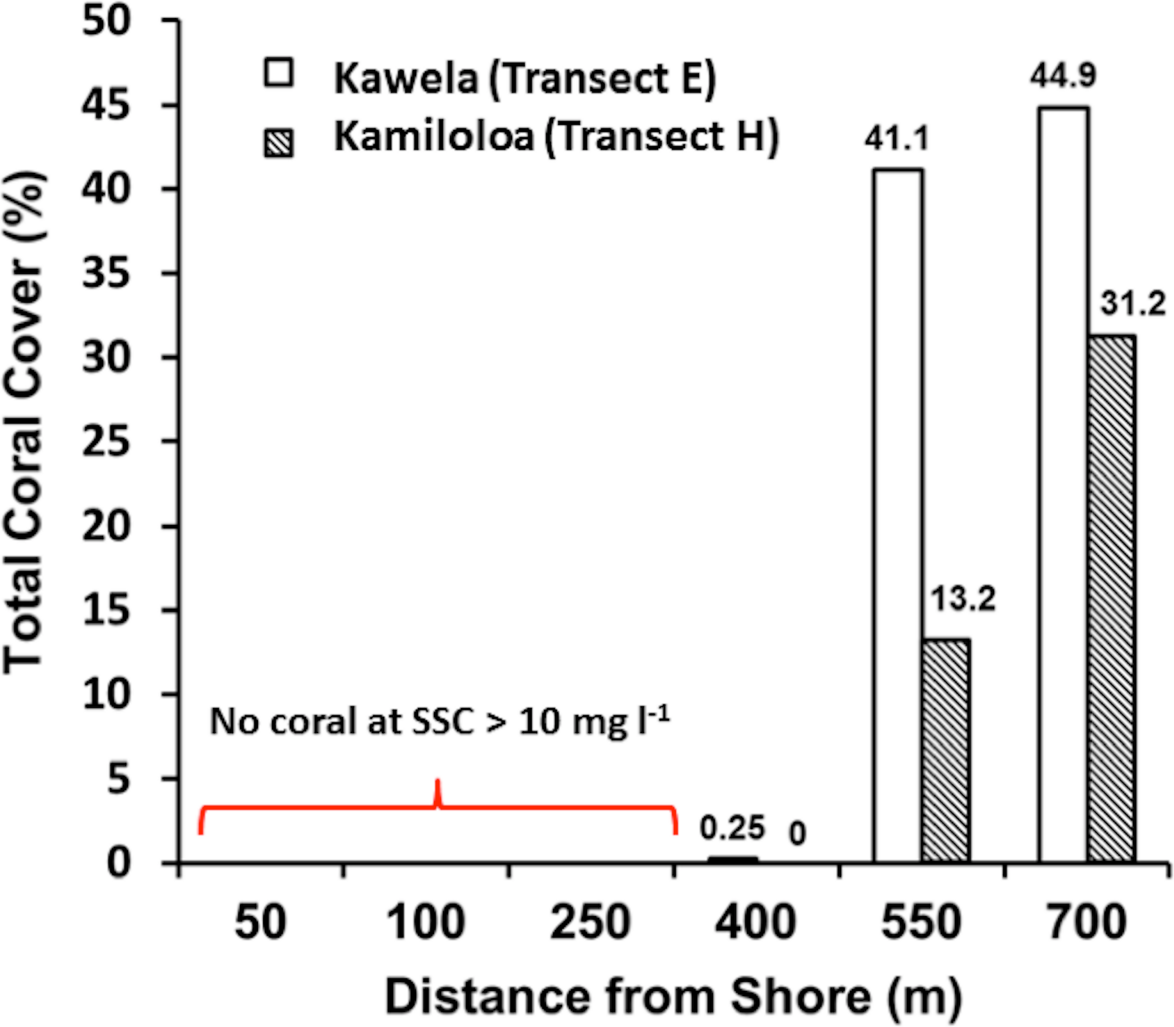
Total coral cover along Kawela (E) and Kamiloloa (H) transects (data from Rodgers et al., 2005).

Transects E (Kawela) and H (Kamiloloa) display the typical pattern along this coast with elevated SSCs inshore that decrease with distance from shore, with SSCs decreasing almost an order of magnitude between the stations 50 m from shore to those 700 m from shore; in general, the transect E stations directly off Kawela Gulch had higher SSCs than those farther west as one moves downcoast along the shore to transect H. These trends are the result of wind-driven waves and currents that continually re-suspend the seabed sediment, causing elevated turbidity and reduced photosynthetically-available radiation as previously reported (Ogston et al., 2004; Presto et al., 2006; Piniak & Storlazzi, 2008). The red dashed line in Fig. 3 shows the 10 mg l^−1^ cutoff, which is the upper SSC boundary for living coral reefs as originally proposed by Rogers (1990). Coral coverage data shown in Fig. 4 supports this generalization, with no coral found at stations that experience SSCs greater than 10 mg l^−1^.

### Mortality

**Figure 5:**
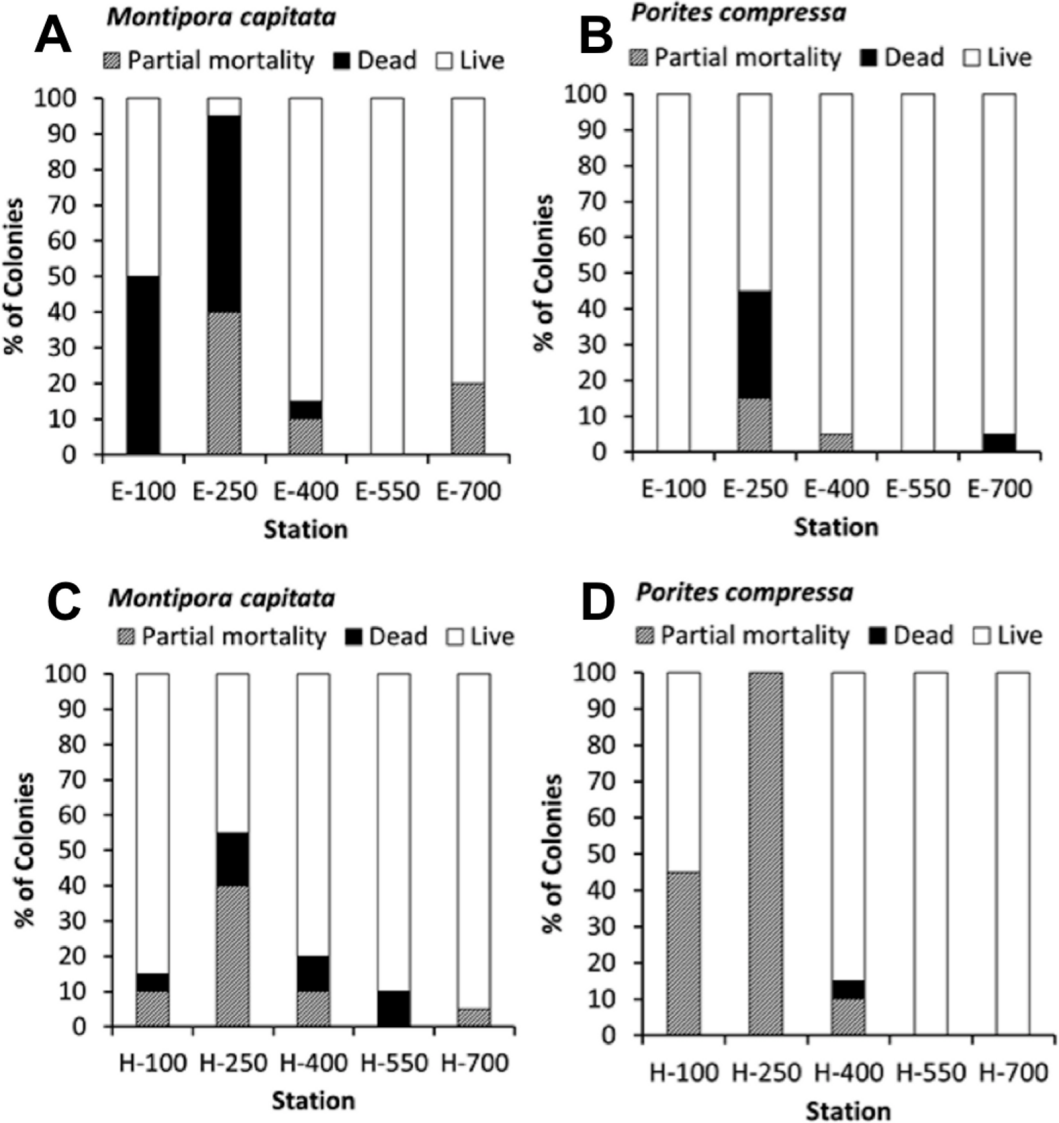
Percent partial mortality and mortality for *Montipora capitata* (A, C) and *Porites compressa* (B, D) (*n* = 400) at all stations along transects E (Kawela) (A, B), and H (Kamiloloa) (C, D).

The percent coral mortality recorded at the end of the 10-month experiment is shown in black with percent partial mortality shown in grey (Fig. 5). Considering the constant level of high turbidity and sedimentation, as described by Field et al. (2008b), coral mortality was remarkably low except at the stations nearer to shore on transect E (E-100, E-250) which is the area closest to a major sediment source at Kawela Gulch. The overall model (*R*^2^ = 60.39%, *p*-value = 0.003) shows that coral mortality/partial mortality decrease with distance from shore, increases with increasing macroalgae and turbidity, and the species *Montipora capitata* had a higher mean mortality/partial mortality than *Porites compressa*. *M. capitata* also showed higher mortality with increasing distance from shore (*p* = 0.05) and with increasing turbidity (*p* = 0.02) than *P. compressa* (Figs. 5A and 5C).

### Growth

**Figure 6:**
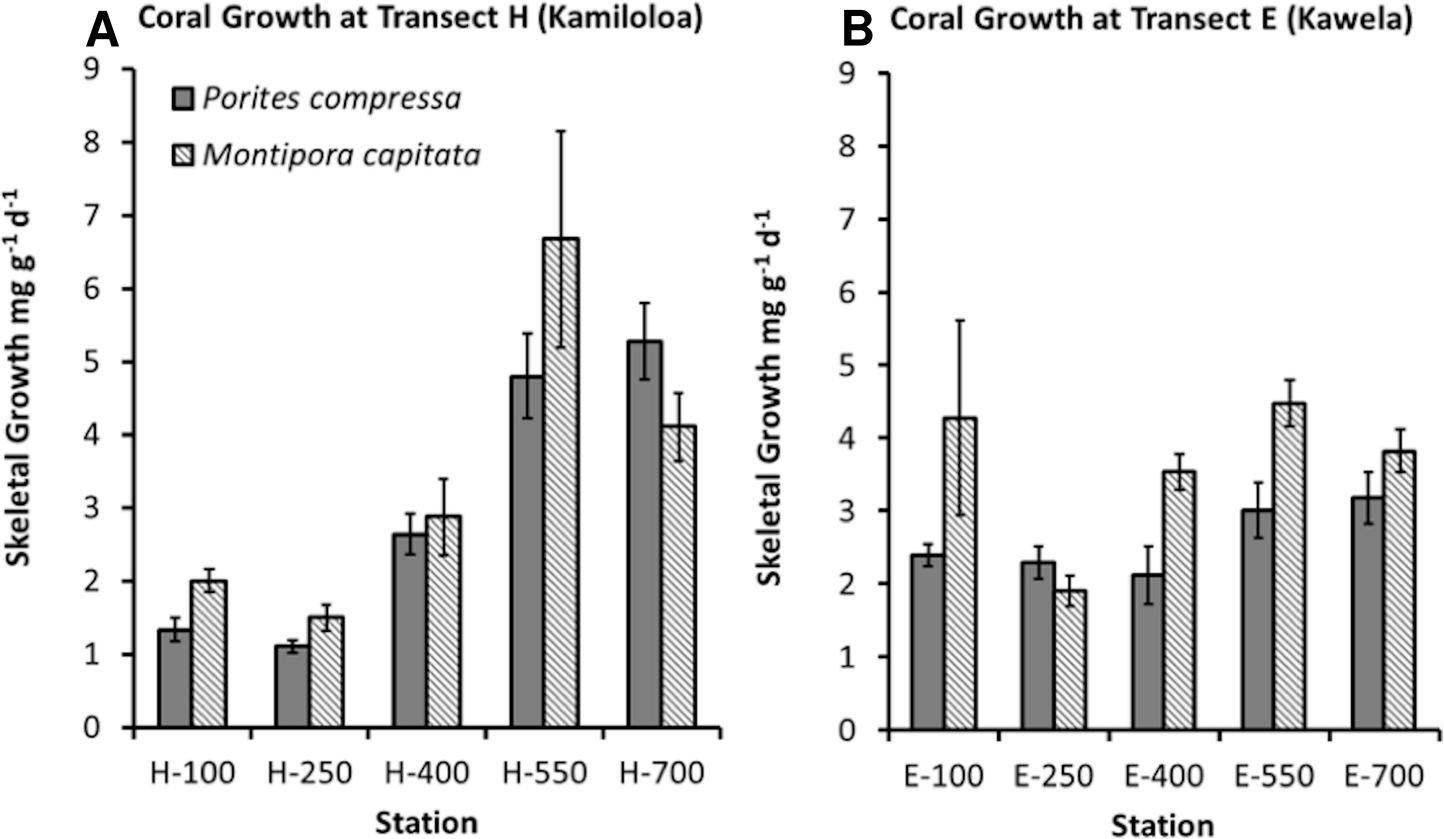
Coral skeletal growth (mg g^−1^ d^−1^) ± SE for corals at all stations along transects H (Kamiloloa) and E (Kawela).

Coral growth was quite good in spite of high SSCs. Coral growth was higher at the offshore sites compared to the inshore sites. The best GLM model using growth as the response variable included species, distance from shore, and benthic cover of macroalgae (*R*^2^ = 66.91%, *p*-value = 0.0001). No difference was found in growth between the two sites. Growth was found to significantly increase with distance from shore (Fig. 6) and decrease with increasing macroalgae. A difference in growth between species was found. *Montipora capitata* shows statistically higher mean growth than *Porites compressa* (*p*-value = 0.001).

Although an overall relationship was found between growth and distance from shore, coral skeletal growth at transect H (Kamiloloa) shown in Fig. 6A shows a strong relationship with increasing distance from shore, whereas coral skeletal growth along transect E (Kawela) shown on Fig. 6B does not show a strong trend. Development of coral reefs at high turbidity levels has previously been reported by Larcombe, Costen & Woolfe (2001), who found that coral assemblages on some inner-shelf habitats on the Great Barrier Reef shelf reach greater than 50% coral cover under extremely high turbidity generated by wave-driven resuspension of sediment. Likewise, Roy & Smith (1971) reported well developed reefs in Fanning Atoll Lagoon in an area where visibility in the water was about 2 m, and suspended load was about 100 times that of the open ocean. The bottom was covered with calcium carbonate mud, where depositional rates appeared to exceed 1 mm/year, and where about 30% of the bottom was covered with live coral. Though there was a decrease in abundance of coral knolls from the clear water areas to the turbid water areas of the lagoon, both areas had lush reef development.

### Impact of macroalgae on coral growth and mortality

At the end of the 10-month growth period, it was apparent that the growth platforms had recruited different amounts of macroalgae. In general linear models, growth and mortality/partial mortality was best explained by macroalgal dry weights. Coral growth was found to decrease with increasing macroalgae dry weights (*p*-value = 0.001) but did not vary with coral species. A statistically significant relationship was also found between coral mortality/partial mortality and macroalgae dry weights (*p*-value = 0.002) where mortality/partial mortality increases with increasing macroalgae and macroalgae increases with distance from shore. Further, there may be a feedback relationship between macroalgae and high SSC. Macroalgae have been shown to have an impact on nearby corals through a variety of mechanisms or combination of mechanisms. These modifications include changes in physical processes of sediment accumulation attenuation of irradiance and marked variation in diurnal dissolved oxygen and pH cycles (Stamski & Field, 2006; Martinez, Smith & Richmond, 2012). On the south Moloka‘i reef flat, sediment trapping by macroalgae has been measured (Stamski & Field, 2006). Macroalgae trapped a mean of 1.26 (±0.91 SD) grams of sediment per gram of dry weight biomass and that sediment was dominantly terrigenous mud (59% by weight). Over 300 metric tons of sediment were estimated to be retained by macroalgae across 5.75 km^2^ of reef flat (54 gm^2^). Macroalgae mats can reduce irradiance by 99% and double sediment accumulation (Martinez, Smith & Richmond, 2012). Algal mats can produce hypoxia and hyperoxia in the extreme diurnal minima and maxima and can significantly acidify the water under the algal mat by decreasing pH and thereby impact corals.

### Recruitment

Extremely low levels of coral recruitment occurred in this experiment (Table 2). Recruitment was from one to three orders of magnitude lower than other reefs measured with the same technique throughout the Hawaiian Islands (Table 3). The few recruits occurred on the seaward coral recruitment tiles, with none in the more turbid areas. No recruitment was found at the three stations nearest to shore at either site. Only one recruit was recorded from Transect E (Kawela) at 550 m from shore and four recruits from transect H (Kamiloloa) at 550 m and 700 m from shore (Table 2).

**Table 2:** Coral settlements at each site ranked in relation to suspended solid concentration (SSC). Only five settlements of three genera were found, with none occurring above 6.1 mg l^−1^ SSC.

Station	SSC (mg l^−1^) ± SE	Total coral settlements	*Montipora*	*Porites*	*Pocillopora*
H-700	3.1 ± 0.4	2		1	1
E-700	3.1 ± 0.4	0			
E-550	3.2 ± 0.7	1	1		
H-400	6.0 ± 1.4	0			
H-550	6.1 ± 1.5	2	1	1	
E-400	7.9 ± 2.1	0			
H-250	10.2 ± 2.8	0			
E-250	23.1 ± 4.7	0			
H-100	24.1 ± 9.8	0			
E-100	36.8 ± 6.0	0			

**Table 3:** Comparison of mean coral recruitment rates (number of recruits m^−2^ year^−1^) from the main Hawaiian Islands measured on standard terracotta coral recruitment tiles.

Site	Mean recruits (recruits m^−2^ yr^−1^)	Range	Reference
Hanalei Bay, Kaua‘i	7,924	403–15,386	Friedlander & Brown (2005)
Puamana, Maui	415	8–1,792	Brown (2004)
Olowalu, Maui	122	95–233	Brown (2004)
Honolua Bay, Maui	41	7–92	Brown (2004)
West Hawai’i	24	0–411	Martin & Walsh (2014)
N. Puako, Hawai’i	167	0–26	Y Stender, pers. com., 2014
Pelekane, Hawai’i	138	0–29	Y Stender, pers. com., 2014
Kawaihae, Hawai’i	304	5–45	Y Stender, pers. com., 2014
Kaunaoa, Hawai’i	282	0–37	Y Stender, pers. com., 2014
**South Moloka‘i**	**5.1**	**0–2**	**This study**

These results are in accord with past coral settlement experiments that have shown a wide variation in coral settlement rates under different conditions. Lewis (1974) reported that a sprinkling of fine washed sand in the bottom of test bowls reduced settlement rate by two thirds. Hodgson (1991) studied coral settlement on a glass surface that was only partially covered with a mixture of sand, silt, and clay. In this situation he found that the larvae will still settle on areas that are clear of sediment. Te (1992) cultured larvae of the coral *Pocillopora damicornis* to four concentrations of sediment (0, 10, 100, 1,000 mg l^−1^) for 14 days under two contrasting water agitation levels and found no significant difference in larval settlement on the glass walls of the containers. Presumably the vertical glass walls did not accumulate sediment, so there was no difference in substrate between the treatments. Babcock & Davies (1991) used a mixture of fine sand and silt of mixed terrigenous and carbonate origin to determine the effect of various rates of sedimentation (0.5–325 mg cm^−2^ d^−1^) on settlement rates of *Acropora millepora* in aquaria. Total number of settled larvae was not significantly affected by sedimentary regime, but higher sedimentation rates reduced coral settlement on horizontal surfaces where sediment could accumulate. The experiments most relevant to the results in Table 2 were conducted by Perez et al. (2014). They measured survival and settlement of *Pocillopora damicornis* larvae on hard surfaces covered with fine-grain (<63 µm) terrestrial silts and clays. Coral larvae were incubated in Petri dishes with different amounts of sediment for two weeks and the percent that settled on the bottom was determined. There was a statistically significant relationship between the amount of sediment and coral recruitment on the bottom, with no recruitment on surfaces having a sediment cover above 0.9 mg cm^−2^, which represents a thickness of 0.05 mm (5 µm). Total survival over the two week settlement tests did not show a significant decline, so the major impact of the sediment was on successful settlement rather than on mortality. The larvae simply would not settle on substrate covered with a thin film of fine-grain terrestrial sediment. The reef flat off south Moloka‘i in the area of the survey is constantly covered with this fine-grain material on all surfaces due to continual re-suspension of seabed sediment. The resuspension creates the elevated SSC conditions. The sediment settles out when insolation-driven winds (“sea breeze”) diminish at night (Field et al., 2008a, and references therein).

## Conclusions

• Corals on the Moloka‘i reef flat can survive and grow in extremely turbid environments (Figs. 5 and 6). Coral growth was negatively correlated with SSC levels, although some growth was documented at SSC levels even in excess of 35 mg l^−1^.
• Coral recruitment was very low (Table 3) in comparison to other areas. Recruitment decreased with increasing SSC and distance from shore (Table 2). No new recruits occurred above a SSC of 6.1 mg l^−1^. We believe that thin coatings of fine-grain terrestrial silts and clays observed on the reef flat effectively blocked new coral recruitment (Perez et al., 2014).
• Presence of macroalgae mats is a major factor controlling coral mortality and growth on the Moloka‘i reef flat.

## Supplemental Information

10.7717/peerj.699/supp-1 Supplemental Information 1 USGS sediment dataset Suspended sediment dataset from the US Geological Society

10.7717/peerj.699/supp-2 Supplemental Information 2 Coral Reef Assessment and Monitoring Program (CRAMP) dataset CRAMP dataset includes coral recruitment, growth, and macroalgal composition.

## Additional Information and Declarations

### Field Study Permissions

The following information was supplied relating to field study approvals (i.e., approving body and any reference numbers): The collection permit was obtained through the Hawaii Department of Land and Natural Resources: Permit SAP 2007.
